# Vascular underpinning of COVID-19

**DOI:** 10.1098/rsob.200208

**Published:** 2020-08-27

**Authors:** Vanessa Wazny, Anthony Siau, Kan Xing Wu, Christine Cheung

**Affiliations:** 1 Lee Kong Chian School of Medicine, Nanyang Technological University Singapore, 59 Nanyang Drive, Singapore 636921, Singapore; 2 Institute of Molecular and Cell Biology, Agency for Science, Technology and Research, 61 Biopolis Drive, Singapore 138673, Singapore

**Keywords:** endothelial dysfunction, virus, vascular biology

## Abstract

COVID-19 management guidelines have largely attributed critically ill patients who develop acute respiratory distress syndrome, to a systemic overproduction of pro-inflammatory cytokines. Cardiovascular dysfunction may also represent a primary phenomenon, with increasing data suggesting that severe COVID-19 reflects a confluence of vascular dysfunction, thrombosis and dysregulated inflammation. Here, we first consolidate the information on localized microvascular inflammation and disordered cytokine release, triggering vessel permeability and prothrombotic conditions that play a central role in perpetuating the pathogenic COVID-19 cascade. Secondly, we seek to clarify the gateways which SARS-CoV-2, the causative COVID-19 virus, uses to enter host vascular cells. Post-mortem examinations of patients' tissues have confirmed direct viral endothelial infection within several organs. While there have been advances in single-cell RNA sequencing, endothelial cells across various vascular beds express low or undetectable levels of those touted SARS-CoV-2 entry factors. Emerging studies postulate alternative pathways and the apicobasal distribution of host cell surface factors could influence endothelial SARS-CoV-2 entry and replication. Finally, we provide experimental considerations such as endothelial polarity, cellular heterogeneity in organoids and shear stress dynamics in designing cellular models to facilitate research on viral-induced endothelial dysfunctions. Understanding the vascular underpinning of COVID-19 pathogenesis is crucial to managing outcomes and mortality.

## Highlights

(1) Pre-existing conditions which compromise vascular health are one of the driving factors for severe COVID-19 outcomes and mortality.
(2) SARS-CoV2 uses multiple entry factors in vascular endothelial cells.
(3) Apicobasal distribution of host cell surface factors and expression changes over the course of the disease could influence SARS-CoV-2 entry and replication.
(4) SARS-CoV-2 infection studies should consider experimental aspects such as polarity of endothelial cells, cellular heterogeneity in organoid models and shear stress dynamics.

## 1. Introduction

Coronavirus disease 2019 (COVID-19) case study reports have called attention to the overrepresentation of cardiovascular diseases, in addition to respiratory diseases, among patients at risk of critical illness and mortality following severe acute respiratory syndrome coronavirus-2 (SARS-CoV-2) infection [1–8]. This susceptibility of infected patients with underlying cardiovascular comorbidities to adverse health outcomes was also common among severe acute respiratory syndrome and Middle East respiratory syndrome—the predecessors of COVID-19 [9,10]. Of note, these comorbidities are often paired with advancing age, thus these individuals already present with compromised innate immunity, impairing their ability to mount an effective immune response following viral infection [11]. SARS-CoV-2 further exacerbates the condition by suppressing the hosts’ innate antiviral defences, associated with low levels of interferon-I and -III and elevated chemokine expression [12].

Initial concerns were also raised regarding the medical treatment of hypertension with adverse COVID-19 outcomes, as studies in animals have shown that the use of renin–angiotensin system blockers–angiotensin-converting enzyme inhibitors and angiotensin receptor blockers result in the upregulation of angiotensin-converting enzyme 2 (ACE2) expression, which is an entry factor for SARS-CoV-2 [13]. Hence, it was speculated that their use may increase susceptibility to and severity of SARS-CoV-2 infection. However, a multinational cohort study analysing electronic medical records of over 1.1 million patients on antihypertensive drugs found no clinically significant increased risk of COVID-19 diagnosis, hospitalization or complications [14]. The direct mechanisms between underlying cardiovascular diseases and COVID-19 morbidity and mortality warrant further investigation.

## 2. The link between compromised vascular health and COVID-19 severe outcomes

The largest case series report to date, of 72 314 COVID-19 cases in mainland China, found that of the 44 672 confirmed cases, 12.8% had hypertension, 5.3% diabetes and 4.2% cardiovascular disease [3]. Moreover, among the 1023 deaths from confirmed cases, 39.7% had hypertension, 22.7% had cardiovascular disease and 19.7% had diabetes [3]. Meta-analysis based on data from seven studies in China, including a total of 1576 cases, found that the most prevalent comorbidities were hypertension (21.1%), diabetes (9.7%) and cardiovascular disease (8.4%) [7]. In addition, severe and non-severe patients were compared to assess the risk of underlying disease with adverse outcome, using a random-effects model to pool odds ratios and 95% confidence intervals. The pooled odds ratio of cardiovascular disease was 3.42 (95% confidence intervals: 1.88–6.22) and hypertension was 2.36 (95% confidence intervals: 1.46–3.83) [7].

In France, clinical characterization of 34 patients admitted to intensive care units with COVID-19-related acute respiratory distress syndrome found that 44.1% had diabetes, 38.2% had hypertension and 8.8% had ischaemic cardiopathy [8]. Furthermore, among these patients, 79.4% had deep vein thrombosis. Collectively, these case reports of confirmed COVID-19 hospitalized patients strongly indicate a strong association between underlying cardiovascular diseases and diabetes with severe health outcomes and fatality following SARS-CoV-2 infection.

Endothelial dysfunction, typically responsible for cardiovascular complications, plays a significant role in the pathogenesis of thrombosis in severe COVID-19 outcomes [15]. Indeed, a significant proportion of intensive care unit-admitted patients with severe COVID-19 develop thrombotic complications [16]. When damaged or dysfunctional, the endothelium releases prothrombotic factors such as von Willebrand factor, predisposing patients to deep vein thrombosis [17]. A pulmonary embolism can subsequently occur when the blood clot travels through the heart to the pulmonary arteries. Here, the vascular occlusion results in heterogeneous pulmonary perfusion and impaired gas exchange, generating hypoxaemia [18]. Both deep vein thrombosis and pulmonary embolism substantially increase a patient's risk of myocardial infarction and stroke [19].

Furthermore, injuries of the pulmonary capillary endothelium increase its permeability, causing fluid leakage into the pulmonary parenchyma [20]. This fluid accumulation overwhelms hydrostatic forces and results in excess flow of fluid into the alveoli. The resulting oedema impairs gas exchange by increasing the alveolar diffusion barrier for oxygen and carbon dioxide [20]. Acute respiratory failure due to the leaky pulmonary capillary has been reported in COVID-19 patients [21].

Interestingly, the Randomised Evaluation of COVID-19 Therapy clinical trial from the University of Oxford (ClinicalTrials.gov, NCT04381936) [22] has shown that treatment with dexamethasone reduced COVID-19 mortality by one-third in patients requiring ventilation, and by one-fifth in patients receiving oxygen therapy [23]. The use of dexamethasone is effective at moderating systemic inflammation [24,25]. Of note, cultured human endothelial cells treated with dexamethasone significantly decreased their endothelial permeability [26]. And, mechanistically, it has been shown in cultured rat brain endothelial cells that this dexamethasone-induced decrease in endothelial permeability is due to the cytoskeletal redistribution and improved continuity of tight junctional proteins [27]. Furthermore, dexamethasone reduces the levels of circulating pro-inflammatory cytokines, such as IL6, TNF-α and IFN-γ [28], which are known to induce expression of endothelial adhesion molecules, such as vascular cell adhesion molecule-1 and intercellular adhesion molecule-1 [29]. Collectively, these may imply that the improved survival of COVID-19 patients treated with dexamethasone may be due to the alleviation of leaky pulmonary capillary syndrome and prothrombotic state.

## 3. Entry of SARS-CoV-2 into vascular endothelial cells: how many gateways?

To date, the pathogenesis and the extent of the damage directly or indirectly affected by SARS-CoV-2 is surfacing with new symptoms reported on a regular basis. SARS-CoV-2 was initially presented as targeting mainly the lung [30]. However, clinical observations and post-mortem findings reported an increasing list of disease presentations that differ from patient to patient [31], indicating that SARS-CoV-2 can infect and damage a wide range of tissue and organs. While both SARS-CoV-2 and its predecessor, SARS-CoV, lead to pulmonary failure, SARS-CoV is mainly a lower respiratory tract disease [32]. *In situ* hybridization study of fatal cases indicate that the primary target cells of SARS-CoV are the pneumocytes and surface enterocytes of the small intestine [33]. Other organs were also reported as positive for SARS-CoV [34], although the relevance of such presence remains debatable. In COVID-19, direct SARS-CoV-2 infection and inflammation of the endothelium was evident across vascular beds [35]. It was postulated that COVID-19 and severe acute respiratory syndrome could also share vascular pathology as there were a few reports of systemic vasculitis in severe acute respiratory syndrome patients [36,37]. Higher fatality rates seen in severe acute respiratory syndrome might have limited its far-reaching impact on extra-pulmonary organs.

Underlying a vascular dysfunction in COVID-19, rare Kawasaki-like multisystem inflammatory characterized by vasculitis—inflammation of blood vessel walls and coronary artery aneurysms—were recently reported in children [38–40]. Furthermore, deadly pulmonary thromboembolism even after virus clearance highlighted that vascular complications inherited from the infection can cause long-term damage (https://www.moh.gov.sg/news-highlights/details/350-more-cases-discharged-344-new-cases-of-covid-19-infection-confirmed). Most of those complications reflect a confluence of vascular dysfunction, thrombosis and dysregulated inflammation [41], supporting the role of endothelial cells as one of the key contributors to the propagation of severe COVID-19 [42].

Successful infection of a host cell by SARS-CoV-2 is a two-step process involving attachment via the receptor and membrane fusion for the release of viral RNA into host cell cytoplasm. Proteolytic activation of the viral spike protein by host proteases has been shown to be essential for the second step [43]. It is widely accepted that SARS-CoV-2 infects host cells using ACE2 for entry and the transmembrane serine protease 2 (TMPRSS2) for spike protein priming [43]. Comprehensive mapping of viral entry gene mRNA using single-cell/nuclei transcriptomic analyses has provided insights into the organs of target during COVID-19 pathogenesis. Transcriptomic analyses of heart samples reported that among the multiple vascular cell types, ACE2 expression is strongest in pericytes, followed by vascular smooth muscle cells (VSMCs), while TMPRSS2 has no detectable or low levels of transcript [44–47]. Although He *et al.* [48] found a number of endothelial cells displaying *Ace2/ACE2* RNA-sequencing counts for the brain and heart of mouse and human tissue, these samples also expressed pericyte markers, implying that the *Ace2/ACE2*-positive endothelial cells were contaminated with pericytes. Challenges associated with the dissociation of pericytes and endothelial cells for single-cell analysis could be responsible for the discrepancy in published data [49]. Those studies along with others that analysed the distribution of the viral entry genes mRNA across multiple healthy human organs highlighted that ACE2 and TMPRSS2 are either poorly or not expressed in the endothelial cells [50,51]. Furthermore, when *ACE2* and *TMPRSS2* mRNA are detected in these endothelial cells, only a scarce number of cells co-express both [52]. On the other hand, endothelial internalization of exosomes originated from the closely associating pericytes has been described [53]. It remains to be explored on the possibility of endothelial infection by exosome-mediated uptake of viral materials from ACE2-expressing pericytes.

In contrast with the transcriptomic data, protein-level analyses of ACE2 seem to suggest endothelial cell expression, in line with previous findings indicating that ACE2 can be post-transcriptionally regulated [54,55]. Immunohistochemical staining of tissue samples from a wide range of human organs revealed ACE2 expression within the arterial and venous endothelial cells [56]. In fact, a strong immunodetection of ACE2 was reported in the endothelium of human tissue samples obtained from the lungs, heart, kidneys, oral mucosa, brain, stomach, small intestine and colon [56–58]. Moreover, histological analysis of lungs obtained on autopsy from COVID-19 patients found an increased number of ACE2-positive capillary endothelial cells, along with severe endothelial injury and disrupted endothelial cell membranes [59], highlighting that the expression of ACE2 may increase during COVID-19 pathogenesis. While ACE2 is readily expressed, immunostaining of human blood vessels indicated that TMPRSS2 is only weakly detected in some endothelial cells [60]; hence, the virus may use alternative host proteases to infect endothelial cells.

**Figure 1.**
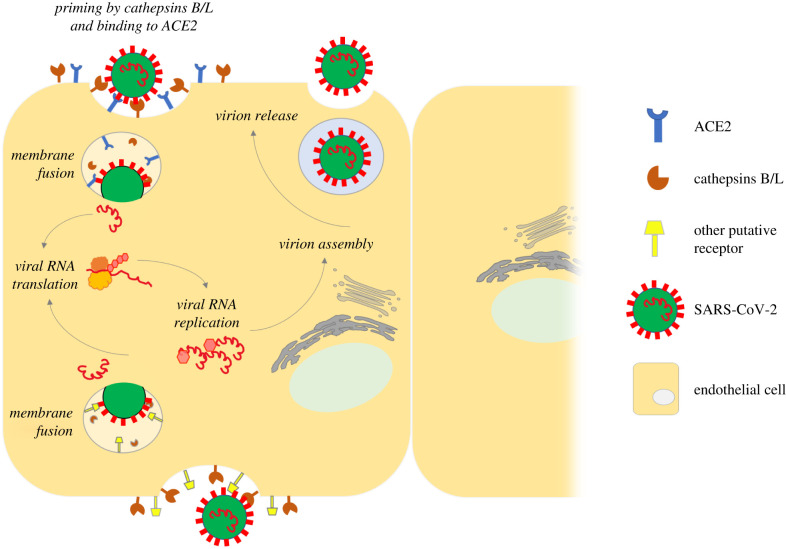
Proposed viral entry mechanisms of SARS-CoV-2 in the endothelial cell. Coronavirus attachment and entry require the presence of known host-factors such as ACE2 and TMPRSS2 and/or cathepsins B/L. Existing data seem to suggest that most of the vascular endothelial cells have an expression profile of ACE2^+^/cathepsins B/L^+^/TMPRSS2^low/−^. In the absence or in the presence of a suboptimal amount of TMPRSS2, cleavage activation of the viral spike protein by cathepsins B/L is crucial for successful membrane fusion and subsequent release of viral RNA into the host cell. The viral RNA is then translated by the host ribosomal machinery to give rise to structural and non-structural viral proteins that are essential for the completion of the virus' replication cycle. SARS-CoV-2 may also use other potential cell surface host factors (e.g. CD147), independent of ACE2, to infect endothelial cells. The subcellular localization of identified receptors and cofactors in the endothelium remains to be determined and will be crucial to the success of establishing endothelial cell models for SARS-CoV-2 infection.

It was previously shown that SARS-CoV-2 can use cysteine proteases cathepsin B/L [43,61] to prime spike protein in TMPRSS2-negative cell lines. These proteases are ubiquitously expressed in the endothelial cells and are involved in vascular remodelling and cardiovascular diseases [62–64]. Here, we propose that the availability of viral entry-associated proteins can plausibly explain the tropism of SARS-CoV-2 for endothelial cells (figure 1). SARS-CoV-2 may invade and spread from the endothelial cell using the ACE2/cathepsin B/L pathway. As the expressions of ACE2 and TMPRSS2 in endothelial cells are upregulated during inflammation, it is also possible that SARS-CoV-2 infects the endothelium via ACE2/TMPRSS2 mechanisms during the course of the disease [65].

Importantly, we cannot rule out the existence of ACE2-independent pathway(s). Recently, CD147, also known as basigin, has been proposed to act as a receptor for SARS-CoV-2. CD147 is a plasma-membrane signalling receptor belonging to the immunoglobulin superfamily, expressed at varying levels on many cell types, including endothelial cells [66] and involved in a wide range of function and diseases along with its interacting partners [67–69]. In addition to its physiological role, CD147 was also shown to be involved in the entry of several viruses. CD147 had been shown to indirectly promote the infection of various viruses including HIV-1 and SARS-CoV through interaction with the host cyclophilin A [70,71]. This chaperone protein [72] is incorporated in the nascent virus particles during infection and redistributed on the surface of the virus [71,73]. *In vitro* results obtained with human cytomegalovirus also reported that CD147 promotes virus entry in endothelial cells, indirectly through other proteins [66]. Treatment with CD147-antagonist peptide 9 has an inhibitory effect on SARS-CoV [71], while the anti-CD147 antibody, meplazumab, was able to block SARS-CoV-2 infection of Vero-E6 cells (monkey kidney epithelial) with an EC_50_ of 24.86 µg ml^−1^ and IC_50_ of 15.16 µg ml^−1^. Importantly, immunoprecipitation, ELISA and surface plasmon resonance supported an interaction between CD147 and the receptor binding domain of viral spike protein. Immuno-electron microscopy also revealed colocalization of viral spike protein with CD147 in viral inclusion bodies of infected cells [74]. To date, more studies are needed to establish whether CD147 can functionally be used as a SARS-CoV-2 receptor. However, a direct interaction of CD147 and SARS-CoV-2 may be of prime interest for therapeutic strategy as it would support that CD147 may act as receptor and promote direct viral entry, unlike for the other viruses. Based on these results, a phase II clinical trial, ‘Clinical study of anti-CD147 humanized meplazumab for injection to treat with 2019-nCoV pneumonia’ (ClinicalTrials.gov Identifier: NCT04275245) [75], to test the inhibitory effect of anti-CD147 antibodies is currently underway. ACE2-independent pathway(s) may also be used to infect ACE2-negative cells. This may be the case in liver vasculatures, where endotheliitis was reported [35] despite the fact that liver sinusoidal endothelial cell do not express ACE2 [50–52,56,58], while the presence of CD147 has been readily reported [76,77]. As expression of CD147 is increased following inflammation and vascular injury [78], this may further increase the infectivity of viruses.

For SARS-CoV-2 to gain a foothold in endothelial infection, it requires further interaction with the host cell machinery. An increasing body of evidence supports a differential interplay between autophagy components and coronavirus. Autophagy is a highly conserved process of cytoplasmic degradation used to maintain cellular homeostasis and eliminate pathogens including viruses. Unwanted elements are enclosed into autophagosomes which eventually fuse with lysosomes to form autolysosome where the degradation occurs [79–81]. However, MERS-CoV has been shown to escape degradation by inhibiting autophagy at the autolysosome formation stage possibly via the viral non-structural protein 6 and accessory proteins 4b and 5, leading to an increase in the number of autophagosomes and a decrease in the autolysosome ratio [82]. Whether this increased number of autophagosomes in turn enhances the viral replication rate as shown for other viruses remains controversial [83]. A converging hypothesis suggested that a subset of autophagosomes generated by the non-canonical ATG5/ATG7-independent autophagy pathway could be used by coronaviruses to carry out their replication [84]. The role of the autophagy pathway during SARS-CoV-2 infection of endothelial cells remains to be determined. A preprint reported that during *in vitro* infection of human bronchial epithelial cells and monkey kidney cell lines, SARS-CoV-2 manipulates the autophagy pathway in a manner similar to MERS-CoV and that pro-autophagic compounds could inhibit viral propagation *in vitro* [85]. Autophagy inducers/modulators are currently under active scrutiny. Many of them are FDA-approved drugs used for other diseases and some have been shown to have inhibitory activity *in vitro* against SARS-CoV-2 [84]. In keeping with this, the combination of autophagy-modulating agents may provide synergistic effects that need to be further confirmed in preclinical studies.

## 4. Role of ACE2 in vasculatures

Of interest, levels of vascular ACE2 expression may be altered in the course of disease. Since ACE2 expression is thought to be indicative of SARS-CoV-2 infection susceptibility, this may have implications for the severity of COVID-19 outcomes. Following partial ligation of the common carotid artery in a murine model of disturbed flow-induced atherosclerosis, endothelial *Ace2* expression significantly increased, but subsequently decreased during disease progression [86]. Thus, given that the endothelial cells display significantly different levels of *Ace2* expression during early and advanced phases of atherosclerosis, this may result in altered levels of endothelial susceptibility to SARS-CoV-2 infection for these individuals. During infection, SARS-CoV infection and its spike protein alone has been found to downregulate ACE2, thus modulating the renin–angiotensin system and contributing to the development of severe lung disease in infected mice [87,88]. In COVID-19 patients, serum levels of angiotensin (Ang) II, the substrate of ACE2, were found to be significantly elevated and positively correlated with pulmonary damage and viral load [89]. However, a post-mortem study of the lungs from COVID-19 victims found a greater number of ACE2-positive cells than control sections [59]. It remains to be determined how SARS-CoV-2 may have disrupted the function of ACE2 in the course of infection, leading to endothelial dysfunction.

ACE2 is an important homeostatic regulator of vascular function, and its altered expression and activity is associated with cardiovascular disorders [90]. In acute lung injury, which can deteriorate into acute respiratory distress syndrome, ACE2 is able alleviate the pathogenesis through protecting pulmonary endothelial cells from apoptosis [91]. Mechanistically, this anti-apoptotic effect of ACE2 is mediated through inhibition of nitric oxide-induced phosphorylation of SMAD2, which prevents phosphorylated SMAD2 from increasing pro-apoptotic proteins and decreasing anti-apoptotic proteins. Human endothelial cell models of atherosclerosis have demonstrated that *ACE2* overexpression alleviates impaired endothelial function, through inhibiting proliferation, and enhancing migration and tube-like formation, indicative of improved endothelial cell function and neovascularization [92]. Further modelling of atherosclerosis has shown that the overexpression of *ACE2* in human endothelial cells can inhibit the inflammatory response by inhibiting endothelial-monocyte adhesion molecules and decreasing Ang II-induced cytokine production [93]. ACE2 has further been proposed to exert a protective role on the endothelial cells in response to shear stress [94]. Exposing cultured human umbilical cord endothelial cells to shear stress upregulated *ACE2* expression, which, through inhibiting proliferation and inflammation, maintained endothelial homeostasis.

Although the role of ACE2 within pericytes remains largely unexplored, lentiviral vector-mediated overexpression of *Ace2* in the rat heart has been shown to protect against hypertension-induced cardiac remodelling by inhibiting perivascular fibrosis [95]. This may imply that ACE2 has a protective role in the perivascular cells, such as pericytes. Within VSMCs, ACE2 functions to protect against Ang II-induced proliferation and migration [92]. Studies found that mice with *Ace2* deficiency had larger vascular lesions in aortic atherosclerotic plaques and arterial neointima formation, due to increased VSMC proliferation [96]. Isolated VSMCs of these mice displayed an augmented pro-inflammatory phenotype. Additionally, aortic VSMCs from *Ace2* knockout mice showed increased NADPH oxidase-dependent reactive oxygen production and apoptosis in response to Ang II [97]. Thus, ACE2 also protects VSMCs from Ang II-mediated vascular inflammation, oxidative stress and cell death. Mechanistically, using human umbilical artery smooth muscle cells, it has been shown that ACE2 protects against Ang II-induced superoxide generation and proliferation by modulation of the JAK2/STAT3/SOCS3 and profilin-1/MAPK signalling pathways [98]. Thus, ACE2, similar to its functions in endothelial cells, plays a protective role in VSMCs. Furthermore, pericytes cells elicit pro-survival effects on endothelial cells [99–101]. If pericytes become damaged by SARS-CoV-2 infection, the loss of pericyte protection would in turn compromise endothelial cell function and vascular integrity.

Taken together, patients with cardiovascular disease may be at increased risk of vascular SARS-CoV-2 infection, and, therefore, severe COVID-19 outcome, depending on the type of vascular complications, and the stage of disease. However, more studies quantifying vascular ACE2 expression during the progression of vascular diseases are needed.

## 5. Endothelial models for studying impact of viral infection

Suitable endothelial cell infection models have great potential for accelerating scientific discovery of COVID-19 pathogenesis. Here, we discuss the experimental considerations in designing endothelial models for infection studies (table 1). At the time of writing, there is a published study on *in vitro* SARS-CoV-2 infection of endothelial cells. Engineered human capillary organoids, generated from patient-induced pluripotent stem cells, were successfully infected with SARS-CoV-2, as confirmed by recovery of viral RNA from organoids post-infection [102]. This model was used to show that SARS-CoV-2 infection can be inhibited by the addition of human recombinant soluble ACE2. Though these organoids lack the natural milieu of the host immune system and surrounding cell types, they do closely resemble human capillaries, containing a lumen, basal membrane, CD31+ endothelial lining and PDGFR+ pericyte coverage. Another study sought to understand why children make up only a small proportion of those experiencing severe COVID-19 complications has been proposed. The research proposition is to expose SARS-CoV-2-infected endothelial cells with plasma from children, healthy adults or adults with underlying vascular disease [112]. To investigate susceptibility to blood clot formation, plasma samples from adults and children with COVID-19 will be used to analyse for protein released by damaged endothelial cells. These findings aim to shed light onto why aged patients, and those with underlying cardiovascular disease, have increased risk of severe COVID-19 outcomes.

**Table 1.** Experimental considerations of viral infection studies in endothelial and epithelial models.

virus	source of cells/animal models	culture format	biological aspect facilitating viral infection	proof of viral infection	effect of infection on endothelial/epithelial cells	other remarks	ref
SARS-CoV-2	human-induced pluripotent stem cells	capillary organoids	closely resemble human capillaries	qRT-PCR quantification of viral RNA	viral RNA release	addition of soluble ACE2 inhibits SARS-CoV-2 infection	[102]
flavivirus	human pulmonary microvascular, microvascular dermal, umbilical vein, brain microvascular, liver sinusoidal microvascular endothelial cells	Transwell inserts	polarized endothelial cells	western blot detection of non-structural protein 1	disruption of endothelial glycocalyx components, hyperpermeability	alteration of permeability occurred in tissue-specific manner, reflecting tissue-specific disease pathology	[103]
reovirus	human brain microvascular endothelial cells	Transwell inserts	polarized brain microvascular endothelial cells, expression of the reovirus receptor predominantly on the apical surface	reovirus antigen-positive cells determined by flow cytometry	monolayer remained intact	polarized release from apical domain	[104]
chikungunya	human brain microvascular endothelial cells	Transwell inserts	polarized endothelial cells	plaque assay, detection of viral antigen by immunofluorescence assay, qRT-PCR quantification of viral RNA	monolayer remained intact	polarized release from apical domain	[105]
influenza	chick embryo	*in vivo* model	polarized endothelial cells, restricted receptor expression	*in situ* hybridization detection of viral RNA	N/A	budding polarity from luminal domain, viral replication not detected in other cell types	[106]
dengue	neonatal mouse cerebrovascular endothelial cells	Transwell inserts	polarized endothelial cells	immunofluorescent detection of viral antigens, qRT-PCR quantification of viral RNA	increase permeability, loss of cobblestone morphology, perturbed tight junction protein localization	infection caused transcriptional upregulation of adhesion molecules and immune mediators	[107]
enterovirus	human intestinal epithelial cells	collagen-coated porous dextran beads	3D polarized model, closely resembles gastrointestinal epithelium, rotating wall vessel bioreactor recapitulates physiological levels of shear stress	immunoblotting for the enterovirus capsid protein, qRT-PCR quantification of viral RNA	morphological changes characteristic of necrosis, such as rounding and membrane lesions	3D cultured cells release more virus than 2D cells at early stages of the viral life cycle	[108]
Ebola	human epithelial adenocarcinoma cells	Transwell inserts	polarized epithelial cells	immunofluorescent detection of viral antigens, qRT-PCR quantification of viral RNA	monolayer remained intact	virus preferentially infects basolateral surface, due to distribution of heparin sulfate	[109]
Japanese encephalitis	human epithelial adenocarcinoma cells	Transwell inserts	polarized epithelial cells	viral titres determined by plaque assays	perturbed tight junction protein localization, hyperpermeability, generation of reactive oxygen species	inhibiting viral replication blocks permeability barrier disruption	[110]
SARS-CoV	human airway epithelial cells	air–liquid interface on collagen-coated porous filters	polarized epithelial cells	qRT-PCR quantification of viral RNA	N/A	polarized release from apical domain	[111]

Numerous models have been designed and developed to study viral infections of the blood vessels, many of which have focused on endothelial polarity. In particular, the apicobasal polarity of brain endothelial cells has been of much interest, given the viral disruption of the blood–brain barrier [113]. Endothelial permeability is usually determined by trans-endothelial electrical resistance, a well-established non-invasive tool for assessing barrier integrity [114], as well as a solute flux assay based on fluorescently labelled dextran to assess macromolecule passage through the polarized endothelial cell monolayer. A study isolated microvessels from mouse cerebral cortex and seeded the cerebrovascular endothelial cells onto Transwell inserts in order to induce polarization [107]. Dengue virus infection of these cells was shown to decrease trans-endothelial resistance and increase macromolecule permeability. Furthermore, dengue infection caused the loss of endothelial cobblestone appearance, and induced changes in subcellular localization of tight junction proteins from membrane to cytoplasm. In another study using polarized human brain microvascular endothelial cells grown on Transwell inserts, chikungunya virus entry and egress were both shown to occur at the apical domain [105]. *In vivo* models such as the chick embryos have also been used to study viral infection [106]. *In situ* hybridization of influenza infected chick embryos revealed that viral RNA was confined to endothelial cells of all organs, and further histochemical analysis showed endothelial-restricted expression of the viral receptor. Additionally, electron microscopy of infected cardiac endothelial cells was used to show that the budding polarity of influenza was only from the luminal side of the polarized vessel, thus preventing the spread of infection into tissue surrounding the endothelium.

*In vitro* modelling of human endothelial cells derived from distinct tissues has shown that the flavivirus non-structural protein 1, from dengue, Zika, West Nile, Japanese encephalitis and yellow fever viruses, binds to endothelial cells and disrupts the endothelial glycocalyx components, triggering hyperpermeability and vascular dysfunction, in a tissue-specific manner [103]. In this model, tissue-specific endothelial cells were seeded on Transwell inserts to achieve apical–basal polarity, and once confluent, treated with the individual recombinant flavivirus non-structural protein 1 proteins. The differential levels of endothelial hyperpermeability correlate with the capacity of non-structural protein 1 to bind to the surface receptors on endothelial cells. Though not directly addressed by the study, these results may suggest that the varied susceptibilities of tissue damage rely on organotypic endothelial expression of viral entry factors.

Bloodstream dissemination within a SARS-CoV-2-infected host is thought to be critical for multiorgan spread and pathogenesis observed in severe cases of COVID-19 [115]. Similarly, mammalian orthoreovirus (reovirus) uses the circulatory system to invade the central nervous system from a distant site of initial infection, by penetrating the endothelial barrier [104]; thus, making reovirus endothelial infection a useful model for systemic viral dissemination. Human brain microvascular endothelial cells, polarized using collagen-coated Transwell membrane inserts, have been shown to be infected by reovirus both apically and basolaterally, though more efficiently on the apical surface due to higher apical distribution of the reovirus receptor JAM-A. Here, no alteration in endothelial permeability indicates that reovirus infection does not alter endothelial barrier integrity. Furthermore, plaque assays on the supernatant showed that the release of reovirus progeny occurred exclusively from the apical surface, regardless of the entry route. In COVID-19 research, nasal and alveolar epithelial cells are generally believed to be the primary sites of viral infection due to the high expressions of SARS-CoV-2 entry factors [51]. Alveolar epithelium shares the basement membrane with capillary endothelium to form the gas-exchange interface. It was then proposed that the basal expression of CD147 in alveolar endothelial cells may mediate invasion of viruses to the bloodstream and extra-pulmonary sites [116]. While CD147 was reported to preferentially localize to the basal surface of epithelial cells [117], its apicobasal distribution in blood vessels has not been established.

Though endothelium polarity is not as well understood as in the epithelium, a lot can be drawn from investigations in apicobasal epithelial polarity, such as the junctional proteins that regulate apicobasal polarity [113]. The infection of SARS-CoV has been modelled in polarized epithelial cells [111]. In this study, polarization was achieved by seeding epithelial cells on an air–liquid interface in collagen-coated porous filters, keeping them separate from the underlying media. Once cells had attached and formed a confluent monolayer, apical medium was removed so that the cells interface in contact with the surrounding air became the apical surface, while the basal surface was in contact with the porous membrane [118]. Immunofluorescence staining and membrane biotinylation showed that ACE2 was more abundantly expressed on the apical surface, thus SARS-CoV also infects epithelial cells using the apical surface [111]. On the other hand, a 3D organotypic intestinal epithelial cell culture model has been established to study enterovirus infection of the gastrointestinal epithelium [108]. In this system, Caco-2 epithelial cells are polarized by using a rotating wall vessel bioreactor that recapitulates physiological levels of shear stress and turbulence. Here, the cells attach to collagen-coated porous dextran beads, and establish apical–basolateral polarity. When compared with 2D cell cultures, which exhibit less complex apical surfaces than the 3D model, the level of intracellular virus production is similar, though viral egress is enhanced in the 3D culture.

Model systems that are representative of *in vivo* infection are important to advance our mechanistic understanding. Though animal models have traditionally been used, they lack the ability to provide controllable experimental conditions. Humanized mouse models, such as the transgenic mice expressing human ACE2, can better recapitulate viral pathogenesis and tropism of SARS-CoV-2 than wild-type mouse strains [119,120]. Human-relevant endothelial *in vitro* infection models have focused on polarity, as this affects both the route of cell infection and release. While the *in vitro* models described here predominantly achieved apicobasal cell polarity using Transwell inserts, polarity can also be achieved by culturing endothelial cells in 3D collagen gels to generate polarized vascular lumens [121]. Of note, in addition to the apicobasal polarity, the endothelium has an added dimension of planar polarity due to blood flow. Shear stress to the cells can be created by the use of parallel plate flow chambers [122]. For the experimental modelling of SAR-CoV-2 infection in endothelial cells, we would like to highlight the relevance of cellular polarity, 3D culture system, recapitulation of complex tissue heterogeneity in organoids and dynamics of shear stresses.

## 6. Conclusion

Even as clinical reports of COVID-19-related vasculopathy grow, there has been little evidence supporting direct infection of the endothelium by SARS-CoV-2 *in vitro*. The failure to recapture these clinical observations in the laboratory could be due to the nature of endothelial monolayer culture used commonly in the laboratory. We propose the use of polarized endothelial cell models for SARS-CoV-2 infection to better reflect the endothelium *in vitro*. These set-ups can help clarify the apicobasal distribution of cell surface factors facilitating SARS-CoV-2 entry and replication.

Basally restricted host factors could explain why apical infection challenge by SAR-CoV-2 is unsuccessful in endothelial cell monolayers *in vitro* while a 3D vascular organoid model was permissive to SARS-CoV-2 infection and replication.

While endothelial dysfunction could have occurred from the ensuing cytokine storm alone, direct infection of endothelial cells by SARS-CoV-2 will suggest a larger role for the vasculature in viraemia and pathogenesis observed in other organs such as heart, liver and kidney. A successful endothelial infection model will allow us to understand the routes of entry and spread for SARS-CoV-2, the extent and types of damage to the blood vessels and the long-term effects of such insults even after virus clearance.

Currently, the long-term impacts of COVID-19 are still unclear. Understanding the extent of damage to the endothelium in COVID-19 will determine if vascular health should be monitored as part of recovery to prevent catastrophic events, especially in patients with underlying vascular conditions. Insights into the nature of endothelial dysfunction in COVID-19 can also better inform the care of patients during active infection and beyond.

## Data accessibility

This article has no additional data.
